# How do machine-generated questions compare to human-generated questions?

**DOI:** 10.1186/s41039-016-0031-7

**Published:** 2016-03-24

**Authors:** Lishan Zhang, Kurt VanLehn

**Affiliations:** School of Computing, Informatics, Decision System Engineering, Arizona State University, Tempe, AZ USA

**Keywords:** Knowledge Base, Biology Student, Atomic Predicate, Question Schema, Seed Pair

## Abstract

Science instructors need questions for use in exams, homework assignments, class discussions, reviews, and other instructional activities. Textbooks never have enough questions, so instructors must find them from other sources or generate their own questions. In order to supply biology instructors with questions for college students in introductory biology classes, two algorithms were developed. One generates questions from a formal representation of photosynthesis knowledge. The other collects biology questions from the web. The questions generated by these two methods were compared to questions from biology textbooks. Human students rated questions for their relevance, fluency, ambiguity, pedagogy, and depth. Questions were also rated by the authors according to the topic of the questions. Although the exact pattern of results depends on analytic assumptions, it appears that there is little difference in the pedagogical benefits of each class, but the questions generated from the knowledge base may be shallower than questions written by professionals. This suggests that all three types of questions may work equally well for helping students to learn.

**Electronic supplementary material:**

The online version of this article (doi:10.1186/s41039-016-0031-7) contains supplementary material, which is available to authorized users.

## Introduction

### Why do instructors need question generation?

In mathematics and similar topics, question generation is widely used perhaps because it is so simple to implement. For instance, an infinite number of questions can be generated from the template “Solve for *x*: [num1]*x* + [num2] = [num3].” where [num1], [num2], and [num3] are variables in the template that should be assigned to numbers. Such question generators are used for generating homework exercises, quizzes, exams, questions to be asked in class, and many other purposes.

In subjects where questions must be answered in natural language, instructors still need a large supply of questions for their homework assignments, quizzes, exams, etc. This paper focuses on technology for generating questions whose answers are in natural language.

### Techniques of question generation

A recent review of educational systems with automatic question generation (Le et al. 2014) noted that there are two major techniques in terms of sources: generating from plain text or from a formal representation of knowledge.

Automatic generation of questions from plain text converts declarative sentences in a text into questions. One of the first systems of this type, AUTOQUEST (Wolfe 1976), helped students check their understanding of readings by generating questions on each sentence. The sentence was first parsed using a natural language parser, the parsed result was matched to a predefined pattern, a template was selected, and a question was generated by filling in variables in the template with values from the pattern. For example, suppose the original sentence was “John bought some fruits.” The corresponding parse tree was:

- (ROOT
  - (S
  - (NP (NNP John))
    - (VP (VBD bought)
    - (NP (DT some) (NNS fruits)))))

When the subject of the sentence is being questioned, the question “Who bought some fruits?” was generated. When the object of the verb phrase was being questioned, the question “What did John buy?” was generated. This general procedure has been used many times (Ali et al. 2010; Heilman and Smith 2009; Kalady et al. 2010; Varga and Ha 2010; Wyse and Piwek 2009). Patterns and templates can be hand authored (Ali et al. 2010; Kalady et al. 2010) or learned from given question-answer pairs (Curto et al. 2012).

The second technique generates questions from a formal representation of knowledge. Perhaps the first system of this type, the SCHOLAR system (Carbonell 1970), represented knowledge as a semantic network and used heuristics for generating questions from it. Later work focused on providing better assessments of students (Lazarinis et al. 2010) and adaptively selecting questions (Dillenbourg and Self 1992). The most recent work (Jouault and Seta 2014) was a history-tutoring system for reviewing the chronology of important events.

Hybrids of the two basic approaches have been explored as well. For instance, Liu and Calvo’s (2012) system first identified key concepts, which were essentially a set of noun phases, in a student’s essay. Next, three types of relationships (Different-to, Similar-to, Kind-of) among the key concepts were extracted from Wikipedia by using Tregex pattern-matching rules on Wikipedia articles. Finally, those relations were used to generate questions based via expert-made templates. Although the system dealt with concepts and relationships, it did not reason with them. The main effort lay in identifying the key concepts and finding the corresponding relations.

Olney et al. (2012) also used a hybrid method to generate questions in biology. Their system first extracted a concept map from texts, where a concept map is a particularly simple type of semantic net. They then used templates to generate questions either from single links in the concept map or from small clusters of specific links. Once again, the emphasis was on extracting knowledge from the text rather than reasoning with the knowledge once it was extracted.

A third hybrid system (Chen 2009) reasoned about knowledge it extracted from text in order to generate questions about the mental states of characters in stories. The story was first transformed to a set of semantic relations in the Scone language (Fahlman 2006). New relations were then inferred by using everyday knowledge (e.g., the definition of “pretend”) already stored in Scone knowledge base so that the mental states could be described more accurately. Finally, yes/no questions were generated to help check readers’ understanding of the characters’ mental states in the story.

Instead of generating questions, another way to quickly obtain a large number of questions is to harvest them from the web. The process is to first get more than enough questions and then rule out the low-quality ones. Section 3 describes how we harvested questions using Google’s keyword search.

### Evaluation of question generation systems

Now that the feasibility of machine-generated questions is established, the next issue is evaluating their quality compared to human-generated questions. Only a few studies have addressed this issue. Liu and Calvo (2012) compared their machine-generated questions to human questions in terms of five quality measures (i.e., correctness, clarity, relevancy, useful for learning concepts, and useful to improve documents) rated by 23 human students. The students were required to write essays at first and then rate the questions that helped to improve their essays. The machine-generated questions were specific to the students’ essays, but the human questions were generic to all the essays. Therefore, this comparison confounded the machine vs. human contrast with the specific vs. generic contrast.

Although comparison of machine-generated questions to human-generated questions are rare, several studies have compared different kinds of machine-generated questions to each other and an evaluation method has come to be widely accepted.

In order to compare questions produced by different question generation teams, Rus et al. (2010) identified five criteria for humans to use in judging the quality of questions: syntactic correctness, ambiguity, relevance, question type, and variety. Although the first two criteria, *syntactic correctness* and *ambiguity*, are probably clear, the other three criteria need explanation. Because the generation task required generating questions from given texts, *relevance* measured the match of the question to its source in the text. Both *question type* and *variety* rewarded systems that generated questions in a variety of formats. For instance, a system that merely prepended “Is it the case that” to every sentence would get low marks for both question type and variety.

Heilman and Smith (2009) came up with similar criteria for ranking the questions generated from Wikipedia and also discussed an important additional issue: over-generation. They listed seven deficiencies that an over-generated question could have. Questions that contained any of them were treated as over-generated questions. Judgments on 644 machine-generated questions were done by three researchers. On average, 87.2 % of the questions were over-generated, but there was a low inter-rater agreement (0.42) according to Fleiss’s *κ*. This work treated over-generation more from a syntactic perspective than a semantic perspective, in part because the machine-generated questions were not assumed to be used for instruction.

The rating systems discussed so far did not directly address the utility of the questions for learning. A pedagogically useful question should prompt deep thought and learning about the domain. For example, most people can correctly answer “yes” when asked “Is a vacuole wall a part of a vacuole?” even when they have never heard of a vacuole. Thus, assessing the learning caused by questions should be viewed as a separate measure from question quality.

Learning gains have sometimes been used to evaluate questions. For instance, Beck et al. (2004) found statistically reliable improvements in reading comprehension after wh-questions. Their study used a within-subject design and logistic regression to predict student’s performance in reading comprehension as a function of the number of wh-questions.

### Research question

Our primary interest was in finding ways to help students learn declarative knowledge domains like biology. Such domains require understanding many concepts and relationships, and answering questions is a common way to exercise and assess such understanding. Unfortunately, the number of questions in textbooks is extremely limited, and teachers usually have little time to invent their own questions. So there is also a need for machine-generated questions in this domain.

Our research question was simple: Are machine-generated biology questions as good as human-generated biology questions according to human judges? We focused on questions for a target population of college students in introductory biology classes.

We assume that questions that are ungrammatical, ambiguous, confusing, or low-quality communication in some other way are probably not going to help students learn biology. However, even if the questions are high quality on these measures, they may or may not help students learn biology. Although we did not measure learning gains in this experiment, we did ask judges to rate the pedagogical benefits of the question. This was intended to be a weak proxy for measuring learning gains.

To do the comparison, we first implemented a method for machine-generating questions from a biology knowledge base. A second set of questions was formed by selecting questions retrieved from the web. The authors of most web questions were probably students and not biology experts. Our third set of questions was comprised of professional human-generated questions from textbooks and a biology study website (http://www.biology-questions-and-answers.com). University biology students were recruited to act as judges. They rated the three sets of questions on four measures. Our primary question was whether the machine-generated questions were worse than the human-generated ones. We were also interested in whether the two types of human-authored questions were of different quality.

In order to keep the project feasible, all questions concerned photosynthesis at an introductory college level. Although the methods could easily have generated more questions, evaluation of the questions was made easier for the human judges by using just one topic and allowing them to review the topic before judging the questions.

The rest of the paper is organized as follow. First, we describe how questions were generated from the biology knowledge base. Second, we describe how questions were collected via web search and how the irrelevant questions were filtered out. Lastly, we describe the comparison of these two types of questions to the professional human-generated questions.

## Generating questions from a semantic knowledge base

Although recent work on question generation has generated semantic knowledge bases from text (Olney et al. 2012), we chose to use an existing knowledge base. Although few such knowledge bases are currently available, the technology and the market for such knowledge bases are increasing steadily (Liu and Singh 2004; Foxvog 2010; “Wikidata”). Thus, we believe that it is only a matter of time before knowledge bases are available that are sufficient for high school basic sciences.

There is no uniform way of structuring a semantic knowledge base, so our methods are limited somewhat to the particular knowledge base used for experimentation. The semantic knowledge base we chose was from Baral and Liang (2012). They used the knowledge base to develop a question-answering system for biology (Baral et al. 2012).

The rest of the section first introduces the knowledge base and then explains how we generated questions from it.

### The knowledge base

The knowledge base distinguishes classes and instances. Classes define an ontology, that is, a hierarchical classification of all that exists. For example, *photosynthesis* is a subclass of *chemical process*, and *chemical process* is a subclass of *event. Event* is one of the root classes, which does not have any parent classes. Another root class is *entity*. For instance, *chloroplast* is a distant subclass of *entit*y. On the other hand, instances represent specific objects or specific events. A triple composed of two instances and one predicate form a relation, which is written as the following format:

- has(photosynthesis001, result, oxygen001)
- has(photosynthesis001, instance_of, photosynthesis)
- has(oxygen001, instance_of, oxygen)

In the first line, *result* is a predicate and *photosynthesis001* and *oxygen001* are instances. The second and third lines indicate that they are instances of the classes *photosynthesis* and *oxygen*, respectively.

There are features common to every instance of a class. For example, any instance of photosynthesis can produce oxygen. These are not properties of the class per se, so a special instance named a prototype instance is used to represent relations common to all members of a class. Any other instance of a class is assumed to have the relations involving the prototype instance of the class as well as the relations in the prototype instances of the super-classes. A class can only have one prototype instance.

The relations directly stored in the knowledge base are called atomic relations. Some relations are inferred during the question generation process, and those are called secondary relations. Similarly, the pre-existed predicates in the knowledge base are called as atomic predicates, and those that are introduced by reasoning only are called secondary predicates. The meanings of atomic predicates are defined in KM (Clark et al. 2004).

### Question generation from seed questions

Our first attempt was to generate questions from a user given “seed” question of the form “What is the difference between *A* and *B*?” The strategy was to design an algorithm that would input a seed question, generate a set of constraints that characterized the relationship between concepts *A* and *B*, then output all pairs of concepts that met the constraints, thus generating more questions of the form “What is the difference between *A* and *B*?” Although we tested the algorithm in biology, the process was domain independent in principle. The rest of this section first introduces the details of the algorithm and then discusses its performance.

#### Preparation

**Fig. 1:**
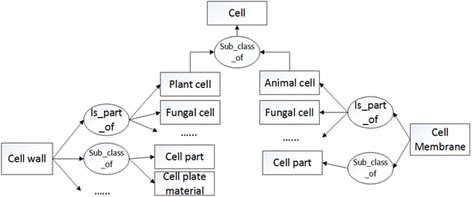
Fragment of the knowledge base. A *rectangle* represents a biology concept and an *oval* represents a predicate

In the knowledge base, concepts are represented as classes and the relations of the concepts are represented in terms of prototype instances, so we extracted all the relations of the prototype instances and directly attached them to the concepts. Figure 1 illustrates the resulting knowledge base.

#### Constraint induction

Our basic approach was based on similarity. Given a user-supplied seed question, such as “What is the difference between *cell walls* and *cell membranes*?”, *A* and *B* comprised a good question if their similarity score (*A*:*B*) was larger than the similarity score of the human-provided seed question concepts (cell wall:cell membrane). Note that there has been many methods developed for calculating semantic similarity, often based on the ontology in WordNet (Banerjee and Pedersen 2002; Budanitsky and Hirst 2001; Jiang and Conrath 1997; Leacock and Chodorow 1998; Lin 1998; Resnik 1995). Although the score we calculated was also called similarity, it was a completely different measure. For instance, *dog* and *hunting dog* are highly semantical related, but the pair was clearly not a good fit for our question template.

We defined two measures. One measured direct similarity and the other measured indirect similarity. As an illustration of these two kinds of similarity, consider Fig. 1. It shows that both *cell wall* and *cell membrane* are part of *Fungal cell*. The direct similarity counts such connections. As an illustration of indirect similarity, note that *cell wall* is part of *plant cell* and *cell membrane* is part of *animal cell* and both *plant cell* and *animal cell* are subclasses of *Cell*. Indirect similarity counts these kinds of relations.

#### Define the direct similarity

The most straightforward way to measure the similarity between two concepts is counting the number of features they have in common and dividing by the average number of features they each have. However, a feature contains both a predicate and an object, and some predicates appear frequently connecting many different objects whereas other predicates appear seldomly. Thus, we calculated similarity on a per-predicate basis and used the resulting vector of predicate-number pairs in the subsequent question generation algorithm.

The formula below is used to calculate the per-predicate similarity:

$$ \begin{array}{c}\hfill \mathrm{similarity}\left(\mathrm{concept}1,\mathrm{concept}2,\mathrm{pred}\right)=\frac{2\times \mathrm{shared}}{\mathrm{total}}\hfill \\ {}\hfill \mathrm{shared}=\left|\mathrm{neighbors}\left(\mathrm{concept}1,\mathrm{pred}\right)\ \cap \mathrm{neighbors}\left(\mathrm{concept}2,\mathrm{pred}\right)\right|\hfill \\ {}\hfill \mathrm{total}=\left|\mathrm{neighbors}\left(\mathrm{concept}1,\mathrm{pred}\right)\right|+\left|\mathrm{neighbors}\left(\mathrm{concept}2,\mathrm{pred}\right)\right|\hfill \\ {}\hfill \mathrm{neighbors}\left(\mathrm{concept},\mathrm{pred}\right)=\left\{x\Big|\mathrm{pred}\left(x,\mathrm{concept}\right)\right\}\hfill \end{array} $$

The similarity value is in the range [0,1], where 1 means that the two concepts are exactly the same in terms of the predicate and 0 means that they have nothing in common.

#### Define the indirect similarity

Indirect similarity means that two concepts are connected by the same predicate to two different objects (e.g., *plant cell* and *animal cell* in Fig. 1) that have the same ancestor (*cell* in Fig. 1). Again, we calculated the portion of shared objects for each shared predicate and stored a vector of predicate-number pairs to represent their indirect similarity. The equation below defines the per-predicate indirect similarity measure:

$$ \begin{array}{c}\hfill \mathrm{indirect}\_\mathrm{similarity}\left(\mathrm{concept}1,\mathrm{concept}2,\mathrm{pred}\right)=\frac{\left|\left(m,n\right)\right|}{\mathrm{pairs}\_\mathrm{total}}\hfill \\ {}\hfill \left(m,n\right)=\left\{m,n\right|m\in \left(\mathrm{neighbors}\left(\mathrm{concept}1,\mathrm{pred}\right),n\in \right(\mathrm{neighbors}\left(\mathrm{concept}2,\mathrm{pred}\right),\hfill \\ {}\hfill m\ne n,\ \exists p\ \mathrm{subclass}\_\mathrm{of}\left(m,\ p\right)\ \&\ \mathrm{subclass}\_\mathrm{of}\left(n,p\right)\Big\}\hfill \\ {}\hfill \mathrm{pairs}\_\mathrm{total}=\left|\mathrm{neighbors}\left(\mathrm{o}\mathrm{b}\mathrm{j}1,\mathrm{pred}\right)\right|\times \left|\mathrm{neighbors}\left(\mathrm{o}\mathrm{b}\mathrm{j}2,\mathrm{pred}\right)\right|,\kern0.5em \mathrm{neighbors}\left(\mathrm{concept},\mathrm{pred}\right)=\left\{x\Big|\mathrm{pred}\left(x,\mathrm{concept}\right)\right\}\hfill \end{array} $$

However, calculating |(*m*,*n*)| is not trivial. A brute force way is for each shared predicate of the two concepts, for each pair of different objects connected to the concepts by that predicate, to enumerate all the ancestors of the two objects and see whether they have an ancestor in common. If they do, a counter is increased by 1; otherwise, the counter stays the same as before. When all pairs for the predicate are explored, the value of the counter is |(*m*,*n*)|.

This algorithm is inefficient because it repeatedly finds all the ancestors for an object. If the same object is visited *x* times, the ancestor set will be generated *x* times. Thus, we cached the ancestor set calculation: When the ancestors of an object are first generated, the set is stored as a secondary property of the object. The ancestor sets turned out to be small enough that this was tractable, so caching reduced the computation from about 17 h to 15 min for generating questions for one seed question.

#### Definition of the constraints

Given two vectors of predicate-number pairs—one that measures the direct similarity of a pair of concepts and the other that measures the indirect similarity—the constraints for “What is the difference between *A* and *B*?” is defined below:

$$ \begin{array}{l}\mathrm{Question}\left(A,B\right)\equiv \\ {}\begin{array}{c}\hfill \forall p\kern0.5em \mathrm{similarity}\left(A,B,p\right)>\mathrm{similarity}\left(\mathrm{seed}1,\mathrm{seed}2,p\right)\hfill \\ {}\hfill \mathrm{and}\hfill \\ {}\hfill \mathrm{indirect}\_\mathrm{similarity}\left(A,B,p\right)>\mathrm{indirect}\_\mathrm{similarity}\left(\mathrm{seed}1,\mathrm{seed}2,p\right)\hfill \end{array}\end{array} $$

In principle, this calculation is run for every possible pair of concepts, *A* and *B*. However, if there are predicates *p* such that *similarity*(*A*,*B*,*p*) is zero but *similarity*(*seed1*,*seed2*,*p*) is non-zero, the calculation does not need to be run because it will be false for this *A*,*B* pair.

#### Automatic schema induction leads to an unstable result

We used eight seed questions to test the algorithm, one for each of these eight pairs: anabolism and catabolism, genotype and phenotype, gill and lung, mitosis and meiosis, nematode and annelid, plasma membrane and cell wall, spore and gamete, and transcription and translation

The algorithm generated an average of 842 questions per seed questions, and the standard deviation was very high: 1721. Several seed questions will be discussed in turn.

The first seed pair was cell wall and plasma membrane. This pair caused generation of eight new questions plus the original seed question. Of the eight new questions, six were reasonable biology questions based on our (limited) experience. Moreover, two of the six questions were asked by someone else before, as confirmed using Google search. The other four did not appear in a Google search, which is interesting. Thus, cell wall and plasma membrane is an example of a good seed question.

The second seed pair was anabolism and catabolism. The generator found no new questions; it generated only the seed question. This occurred because the restrictions generated from the seed question contained some uncommon predicates.

The third seed pair was gill and lung. The generator’s output was 4750 questions. Most of the questions would not make good comparisons. This seed question led to few restrictions, which caused the output of too many pairs of concepts.

The second and third seed pairs are representative of seven out of the eight questions. Except for the cell wall and cell membrane seed pair, all the seed pairs led to either too many or too few generated questions.

These results indicate that the number and the quality of the questions generated from the constraints varied substantially. The variance could be due to the algorithm, the seed questions, and/or the knowledge base. Every seed question is a good comparison question itself. But the knowledge base sometimes did not have adequate descriptions for the concepts appeared in the seed questions, and this led to over-generation. Some concepts in the seed question were attached to uncommon predicates in the knowledge base, and it led to under-generation. It suggested that our method of inducing constraints from seed questions was unstable in part because it depended too strongly on the detailed content of the knowledge base.

These results suggested that any technique that relied on traversing large parts of the knowledge base in an attempt to generate deep, thought-provoking questions was likely to be as fragile as our method. Knowledge bases are just very large and very complex. Nonetheless, instructors still need questions to help their students exercise their knowledge. We decided that some help was probably better than no help, so we decided to focus on generating questions that tapped only small amounts of knowledge. In surveying textbooks, we had noticed that about half their questions were shallow, factual ones (this was confirmed later, as discussed below). This suggested that although instructors probably put a high value on deep questions, they probably also want students to have enough factual knowledge to be able to tackle deep questions, so they probably ask shallow questions as preparation for the deeper ones. If high-quality shallow questions could be generated, then this would remove a load from the instructors and authors, thus allowing them to focus on generating deeper questions. As the remainder of this document demonstrates, even the comparatively modest goal of generating moderately shallow questions proved challenging.

### Preparing for the generation

We choose to study questions about a single topic so that the human judges could more easily refresh their biology knowledge before judging the questions. The chosen topic was photosynthesis, which is a type of event. Thus, we created a subset of the knowledge base that had only knowledge about photosynthesis, its subevents, and concepts that were directly related to such events. For instance, photosynthesis occurs in chloroplasts, so the concept *chloroplast* is included in our reduced knowledge base. A chloroplast is a part of a plant cell, but the concept of plant cell is not in the knowledge base because it is not directly related to photosynthesis or one of its subevents.

Also, the knowledge base was biologically accurate, so it represented details that are irrelevant to students in our target population. For instance, the process of photosynthesis is somewhat different in plants, algae, and photosynthetic prokaryotes. The original knowledge base contained information about all three types of photosynthesis and used three different class names to distinguish them; all were subclasses of the *photosynthesis* class. Some facts about photosynthesis are general to all three types, so the knowledge base uses *photosynthesis* for them. Other facts are specific to plants, so the knowledge base uses the subclass, *photosynthesis_by_plants*. The facts about algae and photosynthetic prokaryotes were removed from the knowledge base so that the knowledge base remains inside the focus of introductory biology textbooks.

In order to simplify the generation of questions, information about instances that would normally be inferred via inheritance from distal prototypes and classes was copied onto the instances themselves. First, features of the prototype instance of a class were copied to all the instances of the class. Second, the transitive closure of the subclass relationship was represented. For instance, given

- sub-class(photosynthesis, chemical process)
- sub-class(chemical_process, event)

the secondary relations

- ancestor(event, photosynthesis)

were added. Other secondary relations were also added and will be described later.

### Question schemas

Questions were generated from question schemas, where a schema is a template with extra information. A question schema consisted of variables, constraints, and question templates. The constraints were matched against the knowledge base in order to bind template variables to concepts in the knowledge base and thus generate a question.

The question schemas depended on the structure of the semantic network we used. As mentioned earlier, the semantic network was extracted from a knowledge base that was used for question-answering purpose, where the entire knowledge base was described in terms of class, entity, event, and relations among these concepts. Since generating questions about classes (e.g., Is photosynthesis an event?) makes no sense in our teaching domain, we focused on generating questions about entities, events, and their relations. Briefly put, we generated “what is” questions to have students learn compositional relations between an event and its subevents. We generated “input and output” questions to have students learn relationships between events belonging to the same parent event. We generated “where” questions to have students learn locational relations between events and entities. We generated “function” questions to have students learn how entities participate in events. This subsection discusses general design issues for the question schemas. Subsequent subsections discuss issues that are specific to the question types just mentioned.

One general issue is that generating all questions of a certain type from the same question schema can be boring to the student. Thus, each question schema had several versions that generated different but synonymous questions, such as “What are the 2 stages of photosynthesis?” and “Could you describe the two sub-processes of photosynthesis?” Also, as mentioned earlier, only facts about plant photosynthesis remained after removing knowledge about two other kinds of photosynthesis. Thus, “photosynthesis” and “photosynthesis by plants” both appear in questions and they have the same meaning. Thus, the knowledge base itself introduces some more-or-less random variation in the generated output that helps prevent boredom.

A difficult problem in natural language generation is generating a referring expression that is both succinct and unambiguous (Carenini and Moore, 1993). In our system, this problem arises when generating expressions that refer to an instance. When an instance fills a slot in a question schema, the name of an instance (e.g., inner_membrane001) cannot be directly used to fill the blank in the question’s template. Although the class of the instance (e.g., inner_membrane) can be used with the underscores removed, and the referring expression is succinct, it is sometimes ambiguous (Carenini and Moore, 1993). For instance, the prototype instance inner_membrane001 represented the inner membrane of a cell whereas inner_membrane002 represented the inner membrane of a chloroplast. Both of them were instances of the class named inner_membrane. If the generator merely used “inner membrane” as the referring expression in the question, then the student may not know which inner membrane was referred to.

Our solution to the problem is simple but far from perfect. In the knowledge base, instances that belong to the same class were distinguishable because they participated in different relations. Including all the relations in the referring expression would certainly distinguish two instances of the same class, but such referring expressions would be too large. Thus, the referring expression generator used only one relation, namely the “part-of” relation. That is, when instance *X* was a part of instance *Y*, the question generator used “the [class of *X*] of the [class of *Y*]” to refer to *X*. If the instance to be referred to was not a part of anything according to the knowledge base, then the question generator used only the name of the class as the referring expression. Moreover, rather than implement this policy as a general subroutine, which inputs an instance and outputs some text, the question templates themselves implemented the policy, and some implemented it slightly differently from others in order to increase the variability of the output. For instance, some templates used “in” instead of “of” (e.g., “the [class of *X*] in the [class of *Y*]”). This solution is clearly not perfect. For instance, it sometimes generates spurious contextualization, such as “the Calvin cycle of photosynthesis.” It also is strongly affected by the reduction of the knowledge base. For instance, it generates “chloroplast” rather than “the chloroplast of the plant cell” because the reduction eliminated the concept plant cell from the knowledge base. Despite the simplicity of this policy, it does an adequate job on a natural language generation problem that is known to be very difficult to solve perfectly (Rus et al., 2010).

Similarly, generating the determiner for a noun phrase is also a difficult problem (Rus et al., 2010). That is, should the referring expression say “chloroplast,” “the chloroplast,” “a chloroplast,” or “any chloroplast”? Our solution is again simple but imperfect. When there is a part-of relationship, then “the” is used twice as the determiner (e.g., “*the* inner membrane of *the* chloroplast”). When such a relationship is absent, then the noun phrase is generated without a determiner (e.g., “___chloroplast”). However, the policy is deliberately implemented slightly different in different question templates in order to increase the variability of the language.

Capitalization (e.g., “the Calvin cycle” vs. “the calvin cycle”) is not handled at all. Our assumption is that capitalization would not affect learning. Indeed, many of the minor disfluencies in the generated questions probably would not affect learning, which is confirmed in the later analysis.

The rest of the section explains how the questions were generated type by type.

### Generating “what is” questions

“What is” questions ask students to explain a concept directly. A simple example is “What is photosynthesis?” Such questions can be answered in many ways, such as explaining the chemical equation of photosynthesis in the student’s own words and pointing out the different stages of the photosynthesis process. The chemical equation actually describes the input and output of the process, which belong to the behavior part of a system. Pointing out the stages refers to the structure part. We decided to have our “what is” questions focus on the structure instead of behavior, because behavior could be handled by other types of questions. Because photosynthesis is an event, all its parts and subparts are also events. Because the evaluation focuses on photosynthesis, rather than cell anatomy, the part-of relations between entities (e.g., a chloroplast is a part of a plant cell) were removed. Thus, our “what is” questions all ask students to describe the different stages of a process.

The key to generating this type of questions is identifying the appropriate concept, which is “photosynthesis” in the example. In principle, any class in the knowledge base can become the questioning concept, but many of them are too general or too specific. For example, it does not make sense to ask “What is chemical process?” As it turns out, neither the too-general nor too-specific concepts have subevents, so generating “what is” questions based only on the part-of relationship rules out those inappropriate concepts.

In order to make it clear that the subevents are to be described, the question schema used the template, such as “What is [the number of sub-events] stages of [the concept].” In order to generate such questions, it is necessary to know the number of subevents of each event. This is pre-computed during the preparation phase and stored as a secondary relationship.

Some events have too many (five or more) subevents defined in the knowledge base. Pointing out the number of events in this case may confuse the students, because different people may have different criteria for distinguishing a subevent. In this case, the question schema does not mention the number and instead uses the following template: “Please explain the process of [concept].”

### Generating “where” questions

“Where” questions are classified into two types. The first one is to question the site of an event. For example, “Where does the Calvin cycle occur?” Generating this type of questions is fairly straightforward. Since *site* is a predefined atomic predicate, the question can be composed by generating a question about its object. As mentioned earlier, simple policies were used for generating referring expressions, so spurious context phrases and articles were sometimes generated. In fact, instead of “where does the Calvin cycle occur?”, the system actually generated “Where does the calvin cycle of photosynthesis occur?”

The second type of “where” question is questioning the origin or the destination of a movement. The knowledge base describes a movement in terms of three atomic predicates. They separately specify the origin, the destination, and the object that is moving. A movement must happen under some circumstance, which means that it must belong to a parent event. So the two corresponding question templates are “In [parent event], where does the [object of the movement] in [destination] come from?” and “In [parent event], where does the [object of the movement] go after it leaves [origin]?”

### Generating “input and output” questions

An “input and output” question focuses on the behavior of a process. The most straight forward “input and output” questions ask what a single process needs for starting and what products it produces. These questions can be easily generated from the knowledge base predicates *raw_material* and *result*. Just as their names indicate, the predicate *raw_material* describes the inputs of a chemical process and the predicate *result* describes its outputs. Again, if the attached process to the predicate is a subevent of another one, the parent event needs to be included to provide the context. Examples of the templates for the input and output questions are, respectively, “What does the [process] require?” and “What does the [process] produce?”

Because the products of one process often serve as the raw materials of another one, we devised questions to help students review these connections. In generating such a question, the generator finds processes that have the connecting relations and then puts them into the templates. If the two processes are the subevents of the same process, the parent process will also be included in the question to give the context. So one question template is “How does [process1] support [process2] in [parent process]?” This question template involves somewhat more knowledge than the others.

### Generating “function” questions

“Function” questions ask about the role of an entity or event in another event. The predicates in the knowledge base corresponding to the role concept are as follows: *enables*, *inhibits*, *causes*, *agent*, *site*, and *raw_material*. Because all these predicates describe how the subject of the predicate (an object) can affect an event, they can all be used to generate a function question. For instance, one question template is “What is the role of [subject] in [event]?”

### Generate the questions in natural language

When the constraints in a question schema are matched against the knowledge base, they bind the variables of the schema. This produces an instance of the schema, such as these:

- What_is(photosynthesis, 2). A “what is” question, with two subevents
- Site_of(calvin_cycle). A “where” question
- Raw_material(photosynthesis). An “input” question
- Role_of(sunlight, light_reaction, photosynthesis). A “function” question

Now that the “question” is written as a relation, we can have several different natural language templates for each type of relation so that students would not get bored when they were answering the same type of questions multiple times. In the process of transforming the relations into the natural language questions, the program randomly selects one natural language template from several predefined ones.

Because the knowledge base was limited to photosynthesis, the generator produced 56 questions: 6 were “what is” questions, 17 were “where” questions, 17 were “input and output” questions, and 16 were “function” questions.

## Collecting questions by Google

**Fig. 2:**
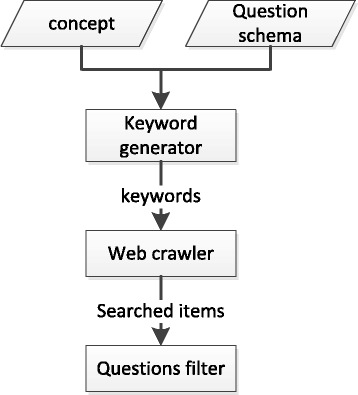
Flow of collecting questions

For a topic like biology, which is widely taught, standard question-answering websites, such as ask.com and yahoo answer, can be used to find student-generated questions. We used a Google custom search engine to search the entire Internet with these question-answering websites prioritized. Figure 2 described how the questions were collected. In order to generate keywords, an algorithm described in Section 3.1 was repeatedly given a concept and one question schema as the inputs. The keywords were then sent to the Google engine, which returned a set of web links. The generator visited those web pages and crawled the relevant text. Questions were selected from the text by the filter described in Section 3.2. In the following, the two main steps were described in detail: generating keywords and filtering out irrelevant items.

### Generating the keywords

Since Google’s search is driven by a list of keywords, it is crucial to choose the keywords properly. To make the collected questions comparable with the machine-generated questions, the keywords need to specify both the concept to be questioned and the type of question. Let us first discuss how keywords for concepts were generated.

An initial list of concepts was extracted from the chapter on photosynthesis in a biology textbook and an article on photosynthesis in Wikipedia. However, the same concept may have several different names that are used by different people and websites. For example, a cell membrane is also called a plasma membrane. Since a keyword search does not know about synonymous terms, the problem needs to be resolved when keywords are generated. To cover all the variations, we used WordNet (Fellbaum, 1998) to augment the initial list of concepts with synonyms.

For generating keywords corresponding to the type of question, we used the keywords shown in Table 1. Any combination of the word in a question-type table and the word in the list of concepts makes one set of keywords. For example, while collecting “output” questions for the concept “photosynthesis,” a possible keyword set is {produce, photosynthesis}. However, a short set of keywords like this often makes Google search return too many non-question sentences. In order to increase the portions of questions retrieved and release part of the burden of the filtering process, we added “what,” “how,” “where,” and “when” to the keyword set to form the entire set of keywords. Thus, there would be four queries that were actually issued for the keyword set {produce, photosynthesis}. They were {what, produce, photosynthesis}, {how, produce, photosynthesis}, {where, produce, photosynthesis}, and {when, produce, photosynthesis}.

**Table 1:** Keywords for Google search

Type	Keywords
What is	what, explain
Where	where, place, site
Input and output	produce, product, result, material, cause, need
Function	function, use, usage, inhibit, enable

### Filtering mechanism

The module web crawler takes the keyword set by set and issues queries via the Google API. The returned search results are in JSON format. Each item contains three properties of the webpage. They are “title,” “snippet,” and “address.” Both the title and snippet can contain a question or parts of a question, so the contents of these two properties are stored as candidates of the final returned questions. The program then goes to the actual web page to get the content in HTML format by the link provided in “address.” The questions may be deeply embedded in the content, so we wrote a parser in terms of HTML tags to extract the possible text and store them as the list of candidates. Specifically, the parser considered the content of every HTML tag as one candidate and left the final decisions to the filters.

Every set of keywords generates a list of candidate questions, and all these candidates are concatenated. The next step is to go through each candidate and check whether it is qualified to be returned. The criteria of the filter are listed below:

- Length: The number of the words in the candidate must be between 3 and 50. A one- or two-word candidate usually is a fragment of a question. A long one is often a piece of JavaScript code.
- Relevance: The candidate must contain one of the concept keywords and one of the question-type keywords.
- Duplication: The candidate should not be too similar to a candidate that is already on the list of qualified questions.

Duplication is defined in terms of the edit distance between the candidate and the qualified question. Different from the usual definition of edit distance, words were used as the atomic units instead of characters. Because absolute value of edit distance is unfair to the items with many words, we divided the absolute value by the length of the current candidate. If the quotient was below 0.2 for all existing qualified questions and the candidate passed the other two measures as well, it would be added to the list of qualified questions.

To improve quality, the questions were further filtered with the Stanford parser. The Stanford parser tagged each word in a sentence with a syntactic symbol. When the first word’s tag was “SBARQ,” which is a sign of a direct question introduced by wh-word or wh-phase, this candidate was judged to be a question. In total, there were eight concepts input to the generator, and the generator finally returned 43 questions.

## Coverage of the machine-generated questions

Unlike many other applications of question generation, it is important for instructional applications that the set of questions that are generated “cover” the target material. In particular, the machine-generated questions were all based on a small number of predicates such as “site” or “cause.” Thus, they could be considered “factual” questions rather than “deep reasoning” questions. Although it would be difficult to precisely characterize the difference between such questions, we can evaluate coverage by counting how many questions are in common between human questions and the machine-generated questions. We assume that if there exist “deep questions” on a topic, then some human will probably ask them. In other words, we need to examine the coverage of the machine-generated questions against human-generated questions.

Since we were working on a narrow domain, “photosynthesis” in entry-level biology, we assumed that the union of the questions collected from textbooks and the questions gathered from the web would serve as an adequately large set of human questions. Given this assumption, we calculated the coverage of the machine-generated questions by matching them against human questions. Some different human questions were actually asking the same thing with different expressions. These questions were combined together. On the other hand, four special “what is” questions such as “What is light reaction process?” were split into several questions, because each of them could be treated from different angles. These “angles” correspond to annotations, which will be described in a moment.

After splitting and merging, there were 59 distinct human questions. Out of the 59 human questions, 24 (41 %) were covered by machine-generated questions and 35 were not. Because one human question sometimes covered more than one machine-generated question, 28 machine-generated questions were covered by the 24 human-made questions. Therefore, of the 56 machine-generated questions, 28 (50 %) matched human questions and 28 did not. The latter figure is important, because it suggests that humans may be failing to generate enough questions to completely cover the knowledge in the knowledge base.

In order to understand the differences between the two sets of questions, we first annotated all the questions according to their topic. We classified topics as structure, behavior, or function, as this three-way distinction is common (Goel et al. 2009) and can be viewed as related to the depth of the question.

Questions asking compositional relations of events, which were sometimes considered as behavior questions, were considered as structure questions here. For example, the question “What are the stages into which photosynthesis is divided?” was treated as a structure question.

Questions annotated with “behavior” were further classified into two levels. Questions asking immediate facts were annotated as “behavior-1,” whereas questions whose answers involved inferences of facts were annotated as “behavior-2.” For example, “What does cyclic photophosphorylation produce?” was annotated as “behavior-1,” and “How do the raw materials of photosynthesis reach the chloroplasts of the leaves?” was annotated as “behavior-2.”

Questions like “What is the function of chloroplast membranes?” were annotated as function questions.

As mentioned before, three “what is” questions were split into ten new questions. Two “what is” questions asked about a process, so they were each split into four questions with the four annotations (structure, behavior-1, behavior-2, and function). The remaining “what is” question asked about an entity. The “behavior” annotation was not suitable for that question, so that question was split into only two ones.

Table 2 describes the results for the four categories of question topics. The details are listed in the Additional file 1: Appendix. The two columns on the right show how many questions were matched by questions from the other category. The number of matched questions differs slightly in the two columns because in a few cases, one human-generated question matched more than one machine-generated question.

**Table 2:** Overlap of machine- and human-generated questions

Category	Machine	Human	Human matched (%)	Machine matched (%)
Structure	9	16	9/16 (56)	9/9 (100)
Behavior-1	30	11	10/11 (91)	13/30 (43)
Behavior-2	2	14	1/14 (7)	1/2 (50)
Function	15	18	4/18 (22)	5/15 (33)
Total	56	59	24/59 (41)	28/56 (50)

For the structure and behavior-1 categories, there was considerable overlap of the machine- and human-generated questions. These are fairly simple questions, so it is not surprising that the two sources tended to generate the same questions. For the behavior-2 and function categories, which tend to include more complex questions, the overlap was much smaller. In terms of a distribution of question types, the machine generated many fewer behavior-2 questions and many more behavior-1 questions than the humans.

This analysis seems to be the first one to study the coverage issue of question generation, so there is no previous work or baseline for comparison. Rus et al. (2007) examined the quality of fact questions generated from plain text by the schemas. Their fact questions were similar to “structure” and “behavior-1” questions in our study. All their generated fact questions were annotated by two human judgers, and a question was classified as “good” when both of the two annotators agreed on that. They found that only 55 % of the machine-generated questions were rated as “good.” Although they were looking at a different measure from us, their work suggests the difficulty of question generation.

## Main study

The research question we wanted to answer was “What are the relative qualities of questions generated from a knowledge base, from searching the web and from a professional source of questions?” For the evaluation, we randomly selected 40 questions generated from the knowledge base, 20 questions from the web search, and 20 from the list of professional questions. There are only 20 questions selected from the web search and professional questions, because the pools of these two types of questions were not big enough to provide 40 random questions. This imbalance would not contribute to any observed results in the later experiment except that the power of the analysis is limited to the smaller groups, *N* = 20. Forty random machine-generated questions were selected because a bigger number of random questions makes a better representation of the corresponding type of questions. These selections formed a photosynthesis question base with 80 questions in total.

The quality of questions was evaluated in terms of four measures: fluency, ambiguity, pedagogy, and depth. Every measure had a scale from 1 to 5. *Fluency* had the judges rate the grammatical correctness. *Ambiguity* asked them whether the question was semantic ambiguous or not. After the judge answered the question, he/she was asked whether answering the question helped him/her understand or review any concepts, which was our measure of *pedagogy*. For *depth*, we asked the judges to rate how much thinking was involved in their answering of the question. The first two measures, fluency and ambiguity, were borrowed from a question generation challenge (Rus et al., 2010).

We did not expect significant differences among the three types of questions in the first two measures, but we expected the professional questions to exceed the other two in *pedagogy* and *depth*. In addition to the quality measures, we also asked the judges to rate the relevance of the question to their level of understanding of biology. So there were five measures for each question in total.

### Procedure

**Fig. 3:**
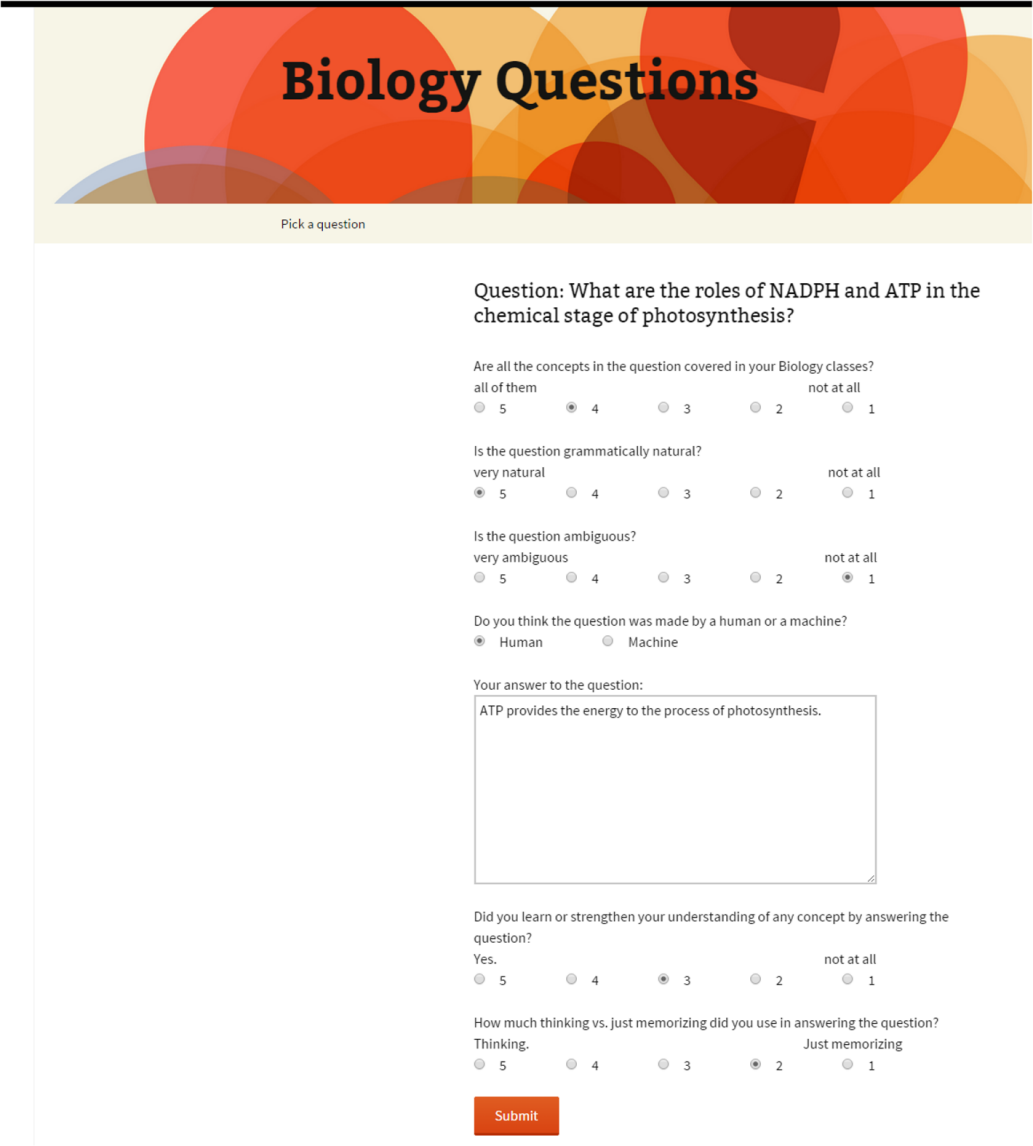
Screenshot of the experiment

All 80 questions were stored in a database, and we implemented a data collection website so that students could answer questions from anywhere and need not come to our lab. The webpage is shown in Fig. 3. To serve as judges, we recruited 12 college students from our school’s Paid Research Participation System, where students registered for participating in research experiments. All but one participant reported that they were native speakers of English; the remaining participant was from India and had been speaking English since she began school. We selected students who reported that they took an intro biology course in the recent two semesters. Every participant was required to answer all 80 questions in order to get 20 dollars as the compensation for finishing the experiment. The experiment was IRB approved.

### Results

Although the questions asked to a student should have been distinct, a technology issue caused some of the early participants to get duplicates of a few questions instead. There 12 judges, and 40 machine-generated questions, 20 web-generated questions, and 20 professionally written questions. Thus, there should have been 12 * (40 + 20 + 20) = 960 data points per measure (e.g., fluency). When the judgments of duplicates were removed, there were 867 data points for each measure.

A typical method for evaluating machine-generated questions is to have two or more people rate every question independently, and the mean of their ratings is treated as the final score of the question. On questions where the scores from the raters were substantially different, the raters discuss and resolve their conflicts. In our experiment, the 12 participants were the raters, but it was impossible for them to discuss and resolve discrepant judgments, so the scores were combined without discussion and adjustment.

We used two different methods for combining scores across judges. The first one assumes judges may be biased, so it normalizes their scores and makes every judge’s mean score for a particular measure be zero with a standard deviation of one. This method assumes that the scores given by the judges form a ratio scale, and thus, it is sensible to take means and standard deviations. The second method assumes that the scales are ordinal instead of ratio, so it is not sensible to take the mean and standard deviation. This method uses medians to compare scores.

### Normalized comparison using the ratio scale assumption

One way to aggregate across the 12 judges is simply to take the means of their scores, as shown in Table 3. Although the scale for every judge was 1 to 5, different judges chose to use different parts of the scale. For example, some judges used mostly 2, 3, and 4 while other judges used the whole scale. Simply taking the mean would give greater weight to the judgments of participants who used a wider range of scores. Thus, as mentioned earlier, we normalized the judges’ scores using a *z*-transformation:

**Table 3:** Mean (and standard deviation) of the original scores

	Knowledge base (*n* = 40)	Web (*n* = 20)	Professional (*n* = 20)
Fluency	4.05 (0.340)	4.06 (0.479)	4.00 (0.341)
Ambiguity	3.97 (0.331)	4.05 (0.367)	4.00 (0.322)
Pedagogy	2.72 (0.342)	2.89 (0.254)	2.94 (0.363)
Depth	2.59 (0.351)	2.66 (0.360)	2.93 (0.478)
Relevance	3.51 (0.460)	3.92 (0.298)	3.65 (0.409)

$$ Z=\frac{\left(\mathrm{score}-\mathrm{mean}\right)}{\mathrm{std}.}+3 $$

where the mean and standard deviation are taken over all 80 scores from a particular student’s judgements about a particular measure (e.g., fluency). By subtracting the mean and adding 3, the *z*-score of every judge/measure combination has a mean of 3, which is the center of the 1–5 scale.

To aggregate across the 12 judges, we simply took the means of their *z*-scores, as shown in Table 4. Reading across the rows suggests that there were few differences among the three different question types. To test that, we ran pair-wise *t* tests. The results are shown in Table 5.

**Table 4:** Mean (and standard deviation) of the normalized scores

	Knowledge base (*n* = 40)	Web (*n* = 20)	Professional (*n* = 20)
Fluency	3.03 (0.343)	2.98 (0.517)	2.97 (0.323)
Ambiguity	2.95 (0.358)	3.05 (0.402)	3.03 (0.291)
Pedagogy	2.90 (0.360)	3.06 (0.281)	3.14 (0.337)
Depth	2.93 (0.319)	2.98 (0.277)	3.16 (0.405)
Relevance	2.86 (0.481)	3.26 (0.334)	3.02 (0.471)

**Table 5:** Pair-by-pair *t* tests of means from Table 4

Relevance	Fluency	Ambiguity	Pedagogy	Depth
Professional vs. knowledge base				
*p* = 0.214	*p* = 0.546	*p* = 0.363	*p* = 0.0167**	*p* = 0.0313**
*d* = 0.344	*d* = −0.163	*d* = 0.235	*d* = 0.669	*d* = 0.669
Power = 0.507	Power = 0.612	Power = 0.517	Power = 0.495	Power = 0.594
Knowledge base vs. web				
*p* < 0.01**	*p* = 0.7153	*p* = 0.3481	*p* = 0.0659*	*p* = 0.5095
*d* = −0.9118	d = 0.1153	d = −0.2709	d = −0.4748	d = −0.1735
Power = 0.360	Power = 0.738	Power = 0.546	Power = 0.448	Power = 0.587
Professional vs. web				
*p* = 0.0765*	*p* = 0.9538	*p* = 0.8453	*p* = 0.4419	*p* = 0.1088
*d* = −0.5472	*d* = −0.0199	*d* = −0.0658	*d* = 0.2383	*d* = 0.4909
Power = 0.579	Power = 0.954	Power = 0.850	Power = 0.590	Power = 0.567

*means *p* < 0.10; **means *p* < .05

The *t* tests did find some significant differences. The web-generated questions were more relevant to students’ knowledge than the other two types of questions. This difference was not surprising because the web-mining method tended to collect questions from students.

Professionally written questions beat the questions generated from the knowledge base in both pedagogy and depth. The questions mined from the web received marginally higher pedagogy ratings than the questions generated from the knowledge base. This is consistent with the analysis of coverage presented earlier which showed that more questions from humans than machines were classified as behavior-2 or function, which seem intuitively to be deeper and potentially more pedagogically useful types of questions.

However, even after normalization, the scores from different raters on the same question and measure were often far apart. This suggests that human judges may be unreliable for those particular measures on those particular questions. Because it was impossible to call back the participants so that they could discuss and agree upon a rating, a second analysis was done discarding the questions/measure combinations with high standard deviation.

For the measure pedagogy, the results are shown in Table 6. Out of the 80 questions, 4 questions were removed because their standard deviations were much bigger than others (more precisely: They were greater than 2 standard deviations larger than the mean of the 80 standard deviations). Among the four questions, there were two from the knowledge base, one from the web, and one from professionals. Table 6 reports the mean values and the significances of the comparisons of the remaining 76 questions. There is still a trend for the professionally written questions to be pedagogically better than the questions generated from the knowledge base, but the trend is only marginally reliable perhaps due to the loss of power due to the loss of data points.

**Table 6:** The comparison of pedagogy after removing questions with high inter-rater variability

	Mean (std.)	vs. Professional	vs. web
Knowledge base	2.96 (0.3128)	*p* = 0.0913*	*p* = 0.1652
		*d* = −0.5005	*d* = −0.3859
		Power = 0.525	Power = 0.490
Web	3.07 (0.2822)	*p* = 0.6696	
		*d* = −0.1396	
		Power = 0.697	
Professional	3.12 (0.3330)		

*means *p* < 0.10

For the measure depth, the results are shown in Table 7. Out of the 80 questions, 2 questions were removed, 1 from the web and 1 from professionals. Removing the two questions did not lead to a different result.

**Table 7:** The comparison of depth after removing questions with high inter-rater variability

	Mean (std.)	vs. Professional	vs. web
Knowledge base	2.59 (0.351)	p = 0.0429**	p = 0.653
		d = −0.577	d = −0.126
		Power = 0.530	Power = 0.685
Web	2.64 (0.354)	p = 0.102	
		d = 0.544	
		Power = 0.505	
Professional	2.83 (0.491)		

**means *p* < 0.05

Although removing question/measure pairs from the sample is one way to cope with unreliability, it reduces power, which makes it harder to tell when two methods of generating questions are truly equal on a measure. Thus, for our second analysis, we used medians instead of means, on the grounds that they are less sensitive to outliers.

### Compare by responses

Among the 867 responses, there were 435 for machine-generated questions, 217 for Google-collected questions, and 215 for textbook questions. Table 8 shows the distribution and median for each measurement.

**Table 8:**
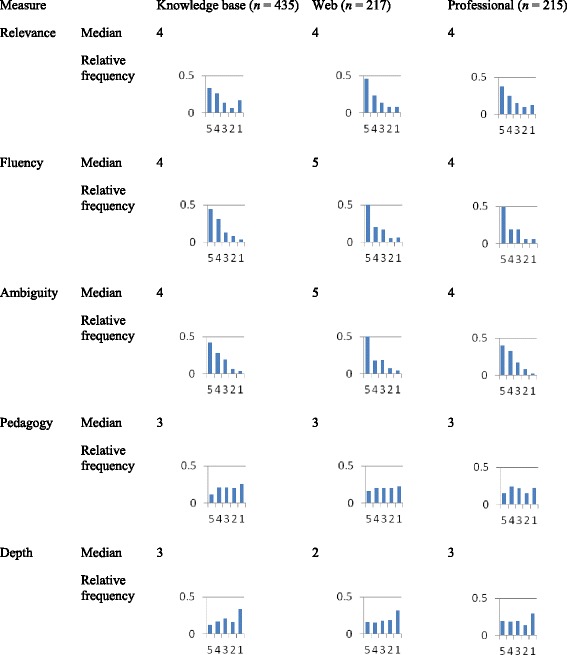
Histograms and median value of the measures

For most measures, the medians were the same. The exception was that the medians of fluency and ambiguity suggest that web-generated questions were better than the other two. However, the underlying distributions for all three question sources on the measure’s relevance, fluency, and ambiguity show that all were similar and all were probably not a major source of pedagogical difficulty.

Pedagogy and depth are the two measures we most care about. The histograms look similar across question sources.

To test whether there any significant differences existed, we ran quantile regression (Koenker, 2005), which is an algorithm to predict the median from the training data. A variable *I* was introduced to represent the identity of the question source, and the significance level of the coefficient of the variable *I* represents the reliability of the difference. For example, in comparing the pedagogy of knowledge base questions to that of web questions, *I* equals 1 when the question is from the web and *I* equals 0 when the question is from the knowledge base. The significance of the coefficient of *I* is the reliability of its difference between the two question sources.

The quantile regression comparisons suggest that the differences in the medians that are apparent in Table 9 can all be trusted. That is, there is no difference between the knowledge base questions and the professional questions on any measure, but there are three differences between the web-mined questions and the other two: The web-mined questions are more fluent, less ambiguous, and shallower than the others.

**Table 9:** Quantile regression comparisons

Relevance	Fluency	Ambiguity	Pedagogy	Depth
Knowledge base vs. professional				
*p* = 1.0	*p* = 1.0	*p* = 1.0	*p* = 1.0	*p* = 1.0
Web vs. knowledge base				
*p* = 1.0	*p* = 0.043**	*p* = 0.042**	*p* = 1.0	*p* = 0.016**
Web vs. professional				
*p* = 1.0	*p* = 0.012**	*p* = 0.047**	*p* = 1.0	*p* = 0.064*

*means *p* < 0.10; **means *p* < 0.05

The pattern of median results is not consistent with the pattern of results from the means of the *z*-scores. The latter showed that the knowledge base questions were shallower and less pedagogically beneficial than the professional questions and that the web-based questions were in between the other two types on all measures except relevance, where they were ranked higher than both the professional and knowledge base questions.

These conflicting results leave us with an interpretation challenge. The main issue is how to interpret the conflicting results for pedagogy and depth. The histograms of Table 8 suggest that the judgements were nearly uniformly distributed, which in turn suggests that medians may not be sufficiently sensitive to detect differences. Thus, we tend to put more confidence in the results about pedagogy and depth that are shown in Tables 6 and 7, which factor out the influence of questions with high inter-rater variability. We can probably ignore the conflict about fluency, ambiguity, and relevance because those are not the main measures of interest. Moreover, we had informal interviews with some of the participants after the study, and no one claimed that they had trouble in understanding the questions. So fluency and ambiguity probably would have little effects on students’ learning. The next analysis addresses this question.

### Correlation of the measures

If the goal of generating a question is to enhance students’ learning, is relevance, fluency, ambiguity, or depth of the question going to affect the pedagogy? To answer this and similar questions, we calculated the correlations of the measures. The original 867 responses per measure were used without aggregating or normalizing or dividing by question source.

The result was showed on Table 10. From the table, the depth of the question is highly correlated with the pedagogy. All the other three measures have very low correlations with pedagogy. Relevance, fluency, and ambiguity are moderately inter-correlated and apparently uncorrelated with depth and pedagogy.

**Table 10:** Pearson’s correlation of the different features of the questions

	Relevance	Fluency	Ambiguity	Pedagogy	Depth
Relevance		0.398	0.331	0.168	0.140
Fluency			0.328	0.202	0.124
Ambiguity				0.061	−0.013
Pedagogy					0.800
Depth					

These results are consistent with those of Table 9. The web-mined questions received high scores in fluency and ambiguity but are tied with or even worse than questions from the knowledge base and professionals in pedagogy and depth. This suggests that as long as the question itself is understandable (staying at the relatively high level in fluency and ambiguity), its pedagogical features are unrelated to the syntactic aspects.

**Fig. 4:**
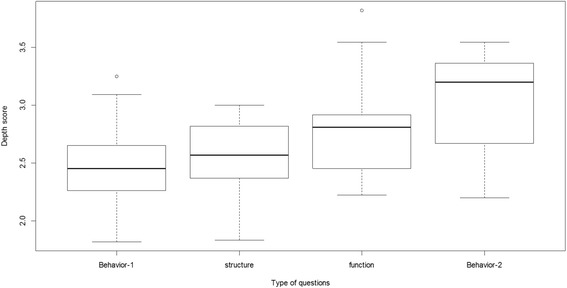
Mean of depth for each type of annotations

When we measured the overlap of the human-generated questions and the machine-generated questions, we used four annotations: structure, behavior-1, behavior-2, and function. It seems intuitively clear that behavior-2 and function questions are deeper than the other two types. To explore whether students agreed with this intuition, each of the 80 questions used in the experiment were annotated with “behavior-1,” “behavior-2,” “function,” or “structure” as described in Section 4, except for two “what is” questions that could not be annotated with a single flag. So 78 questions were split into four groups based on the types of annotations. As Fig. 4 shows, questions with different annotations have clearly different depth score means. An ANOVA was conducted to confirm our assumption (*p* < 0.001, *F* = 6.414, df = 74). A Tukey HSD test was performed to test the group-by-group differences. If behavior-2 and function questions are considered as deep questions, then both should get higher scores than behavior-1 and structure questions. Both behavior-2 and function questions do have significantly higher scores than behavior-1 questions. But only behavior-2 questions have a significant higher depth score than structure questions. It is probably because some function questions have very obvious answers. For example, “What do chloroplasts enable plant cells to do?” can be simply answered as “photosynthesis,” whereas behavior-2 questions were defined such that their answers always required inference.

The analysis was repeated with just two categories, shallow and deep. That is, behavior-2 and function questions were aggregated together as the deep group, whose mean depth score was 2.85 (SD = 0.41, *n* = 35), and behavior-1 and structure questions were grouped together as the shallow group, whose mean of the depth score is 2.51 (SD = 0.32, *n* = 43). The difference is significant according to *t* test (*t* = 3.948, *p* < 0.001), with a large effect size (Cohen’s *d* = 0.92). This suggests again that our coding of questions corresponded to the depth judgments of the 12 raters.

## Discussion

In order to compare questions generated from the knowledge base, mined from the web, and collected from textbooks, we first examined the coverage of the human-generated questions (the latter two categories) by the questions generated from the knowledge base. As expected, the machine-generated questions had a much higher coverage of shallow human questions than deep human questions. This is not a surprise because each question generation schema used with the knowledge base only accessed a small number of knowledge base relationship. We tried generating complex question schema using seed questions to infer the pattern of knowledge base relationships for the schema. This turned out to be too sensitive to the details of the knowledge base. Some seed questions generated far too many question schemas for a human author to check, and other seed questions generated too few candidates. Therefore, when questions are generated from a knowledge base, it seems that a practical method is to have a human write the constraints for generation.

In terms of the quality of the machine-generated question, our experiment shows that the questions from the three different sources have only minor differences in their quality ratings. Although some significant differences in their fluency and ambiguity ratings were observed, the differences in pedagogical and depth ratings were unreliable, appearing only with some statistical tests and not others. In particular, we found:

- Knowledge base vs. professional:
  - *z*-scores: for *pedagogy* and *depth*, knowledge base < professional
  - *z*-scores with removal of questions with high inter-rater variability: no reliable difference on *pedagogy*, reliable difference on *depth*
  - Medians: no reliable differences
- Knowledge base vs. web mining:
  - *z*-scores: for relevance, knowledge base < web
  - *z*-scores with removal of questions with high inter-rater variability: no reliable differences on *pedagogy and depth*
  - Medians: for fluency and ambiguity, knowledge base < web; for *depth*, knowledge base > web
- Web mining vs. professional:
  - *z*-scores: no reliable differences (for relevance, web > professional with marginal significance)
  - *z*-scores with removal of questions with high inter-rater variability: no reliable differences on *pedagogy and depth*
  - Medians: for fluency and ambiguity, web > professional

If these ratings from 12 biology students can be trusted as proxies for the pedagogical quality of the questions, it appears that all three sources are equally beneficial on the whole.

However, a different analysis did show a difference in the depth. Unlike the main evaluation, which was conducted on a sample of each type of questions, this analysis compared all the machine-generated questions to all the human-generated questions, both professional and web-mined, merged together. When we classified questions as shallow (both structural and behavior-1) vs. deep (both behavior-2 and function), we found strong correlations with the judgements of the 12 biology students but the ratings systems were not identical. Using our classifications, we found that about 50 % of the human questions were deep whereas only about 30 % of the machine questions were deep. Moreover, only 15 % of the human deep questions were also generated by the machine, while 70 % of the human shallow questions were generated by the machine. These figures are not terribly surprising, as they are consistent with the design goals for the machine-generated questions. However, we were pleasantly surprised to see that the machine generated a very high percentage of the shallow questions that humans generated.

## Conclusion

Given a knowledge base, we developed a rule-based algorithm to generate biology questions and developed a method to collect biology questions by searching the web with keywords. When 12 biology students judged the pedagogical merit, depth, relevance, fluency, and ambiguity of the two different types of questions to professional human-made questions, the pattern of results depended on whether we assumed the judgements were a ratio scale or an ordinal scale. If we assumed a ratio scale, then professionally generated questions were rated as deeper and more pedagogically beneficial than questions generated from the knowledge base but there were no differences in fluency and ambiguity. On the other hand, if we assumed an ordinal scale, then there were differences in fluency and ambiguity but no differences in depth or pedagogy except that depth of the questions from the web was smaller than that of the machine-generated questions. Correlations of the measures suggest that depth and pedagogical merit were highly correlated but no other pair of measures were.

However, when we classified the questions by their topic, there were differences due to the design of our question schemas, in that about 50 % of the human-generated questions addressed deep topics (function and behavior-2) whereas only about 30 % of the machine-generated questions did. However, the machine-generated questions did cover a large fraction of the shallow human-generated questions. Conversely, only 50 % of the machine-generated questions were covered by human-generated questions.

This suggests that machine-generated questions can play an important role in instruction in that they can generate the shallow questions that human authors would otherwise need to generate. This would leave human authors to focus on generating deep questions. Moreover, if our method for collecting human-generated questions is a fair representation of questions that humans generate, then it appears that humans are failing to generate about half the shallow questions that students need. Thus, this suggests that machines may be better than humans in that they can generate a more complete set of shallow questions.

However, the results also sound a cautionary note, in that the biology students did not perceive much difference in the depth or in the pedagogical benefits of the different types of questions. It is not clear whether our classifications or their judgements are better predictors of the actual pedagogical value of the questions. One possibility is that the biology students tend to interpret a question as a beneficial one when they are unsure about the answer no matter whether the answer can be easily learned (shallow questions) or not (deep questions). If the biology students are correct that even shallow questions are beneficial if one does not know the answer, then the machine-generated questions should be as good as the human-generated ones at least for the beginners to get familiar with a subject like biology. We actually have already built a simple tutoring system that help non-biology majors students learn photosynthesis with the machine-generated questions and found a significant difference between pre-test and post-test scores.

Another caution concerns the generality of the question generation schemas, which must match the structure of the knowledge base. If a new knowledge base were used with a different structure, new questions schemas would be needed.

Moreover, the knowledge base can be considered to be a model of an ideal student’s knowledge (Carbonell, 1970), so depending on the learning objectives defined by different instructors, the knowledge base may need to be adjusted correspondingly. For example, biology teachers in high school do not expect their students know as much about photosynthesis as college students who major in biology. While *removing* knowledge to change a college knowledge base into a high school one would not require changing the question schemas, *adding* more knowledge may bring in new predicates and thus necessitate new question schemas.

## Additional file

Additional file 1: Appendix. Details of the four categories of question topics. (PDF 76 kb)
