# Supplementary material for: How do machine-generated questions compare to human-generated questions?
Source: Res Pract Technol Enhanc Learn. 2016 Mar 24;11(1):7. doi: 10.1186/s41039-016-0031-7 (PMC6302853; doi:10.1186/s41039-016-0031-7)
Supplement: Supplementary file 1 — Appendix. Details of the four categories of question topics. (PDF 76 kb) [file 41039_2016_31_MOESM1_ESM.pdf]

Appendix:

**Human questions**	**Answer**	**Machine questions**	**Type**
What are chloroplasts?<br>-structure	Organelle in cell		Structure:<br>boundary
What are chloroplasts?<br>-function	Site of photosynthesis		function
What is light reaction process?<br>-structure	It consist of cyclic photophosphorylation and noncyclic photophosphorylation	What are the 2 stages of the light reaction in photosynthesis?	Structure
What is light reaction process?<br>-behavior-1	Light reaction takes water and sunlight as reactants and produces Oxygen, ATP and NADPH	What is/are the product(s) of the light reaction in photosynthesis?<br>What does the light reaction in photosynthesis require?	Behavior-1
What is light reaction process?<br>-behavior-2	It produces ATP and NADPH, both of which are reactants of Calvin cycle.		Behavior-2
What are dark and light reactions in photosynthesis?<br>What is light dependent and light independent reactions in photosynthesis?	Light reaction and Calvin cycle are the two stages of photosynthesis.	What are the 2 stages of photosynthesis?	Structure
What is cyclic photophosphorylation?<br>-structure	Chlorophyll first absorbs photons to make photosystem I release	Could you describe the three sub-process involved in the cyclic photophosphorylation	Structure

	electrons. The electrons go through ferredoxin, cytochrome b, plastocyanin, and go back to photosystem I.	of light reaction?	
What is cyclic photophosphorylation? -behavior-2	It produces ATP, which could be used in Calvin cycle.		Behavior-2
Where does photosynthesis occur in a plant?	chloroplasts	Where does photosynthesis by plant happen?	Structure
In which specific part of a leaf cell does photosynthesis takes place?			
What are the site of photosynthesis?			
Where is the chloroplast located?	Plant cell		Structure: boundary
In which part of the chloroplast does photosynthesis take place in?	Light reaction takes place in the thylakoid membranes, and Calvin cycle takes place in the stroma		Structure: boundary
What is the fluid part of the chloroplasts where the calvin cycle takes place?	stroma	Where does the calvin cycle of photosynthesis occur?	Structure
Where do the enzymatic reactions of the calvin cycle			

take place?			
Where do light reactions occur?	thylakoid membranes		Structure: boundary
In what part of photosynthesis sugar is produced?	Calvin cycle produces sugar	What is/are the product(s) of the calvin cycle in photosynthesis?	Behavior-1
In which part of photosynthesis is oxygen produced?	Light reaction produces oxygen	What is/are the product(s) of the light reaction in photosynthesis?	Behavior-1
What molecules are the reactants and products of photosynthesis?	The reactants of photosynthesis are water, CO_2, sunlight. The products of photosynthesis are oxygen, sugar	What does the photosynthesis need?<br>What does the photosynthesis produce?	Behavior-1
What are the materials required by multicellular organisms for the processes of respiration and photosynthesis?	Respiration requires sugar and oxygen.<br>Photosynthesis requires sunlight, CO_2 and water	What does the cellular respiration need?<br>What does the photosynthesis need?	Behavior-1
What are the raw materials of photosynthesis?	Photosynthesis requires sunlight, CO_2 and water	What does the photosynthesis need?	Behavior-1
What are the four basic ingredients needed for photosynthesis?			
What are the raw materials of photosynthesis and its role?			
What are the three main			

limiting factors of photosynthesis?			
What is the energy molecule produced in the mitochondria and chloroplast?	ATP		Behavior-2
How do the raw materials of photosynthesis reach the chloroplasts of the leaves?	Water is absorbed through the root hair then into the xylem of the roots and into the xylem of the stem, it then goes through the xylem of the leaves into the mesophyll cells and finally into the chloroplasts.<br>Carbon dioxide diffuses from the atmosphere through the stomata and then into the intercellular airspaces in the leaves and finally into the chloroplasts of the mesophyll cells.<br>The chlorophyll and other pigments in the thylakiod membrane absorb the solar energy to drive photosynthesis.		Behavior-2
What does the light reaction in	It produces oxygen, ATP	What is/are the product(s) of	Behavior-1

photosynthesis produce?	and NADPH	the light reaction in photosynthesis?	
What molecules produced during the light reaction of photosynthesis are needed to carry out the dark reaction?	Light reaction produces ATP and NADPH, both of which are used in Calvin cycle.	How does the light reaction support the calvin cycle?	Function
Why a toxin that inhibits an enzyme of the calvin cycle will also inhibit the light reactions?			
Which are the subproducts of the photochemical stage that are essential for the chemical stage of photosynthesis?			
What does the light reactions of photosynthesis supply the Calvin cycle?			
What is light reaction process? -function			
What are two products in photosynthesis of the light reaction that are used for the dark reaction?			

Where does the energy used to produce atp in the light reactions of photosynthesis come from?	Sunlight		Behavior-2
What does the calvin cycle use to produce high- energy sugars?	ATP and NADPH		Behavior-2
What else is needed besides water for the calvin cycle to take place?	CO2	What is the raw material of calvin cycle in photosynthesis?	Behavior-1
What does cyclic photophosphorylation produce?	ATP	What does the cyclic photophosphorylation in light reaction produce?	Behavior-1
What is cyclic photophosphorylation? -behavior-1			
What does non-cyclic photophosphorylation produce?	ATP, Oxygen and NADPH	What does the noncyclic photophosphorylation in light reaction produce?	Behavior-1
What is the function of the chloroplast?<br>What is the chloroplast function?	They are the main site of photosynthesis in plant cells and help convert energy from the sun into		Function

What is the function of the chloroplast in plant cells?<br>What are chloroplasts and what is their role?<br>What does chloroplasts enable plant cells to do?	sugars for the plant.		
What is the function of chloroplast membranes?	The outer membrane is permeable to small organic molecules, whereas the inner membrane is less permeable and studded with transport proteins. The innermost matrix of chloroplasts, called the stroma, contains metabolic enzymes and multiple copies of the chloroplastgenome.		Function
What do you think will happen if you insert chloroplast into animal cells in humans?	Animal may also perform photosynthesis.		Function
What is the role of light in the light reactions?	The light<br>reactions use light energy to produce ATP and NADPH.	What is the role of the sunlight in the light reaction of photosynthesis?	function
What is the primary function of the calvin cycle in green plant?	Construct simple sugars from carbon dioxide (CO_2).		function
What is the main function of cyclic photophosphorylation?	The function of cyclic photophosphorylation is to produce ATP.		Function
What is cyclic photophosphorylation?<br>-function			

Why do plants need chloroplast and the animal cells don't need it?	Animals can acquire glucose by eating plants, but plants have to produce glucose by themselves.		Function
What would happen to photosynthesis if all three carbon sugar compounds produced in calvin cycle were used to make organic compounds?	The cycle would stop. You need to reinvest these compounds into the cycle to keep the biochemical process moving.		Function
How is light from the sun transformed into chemical energy to be used by the living beings on earth?	Light from the sun is transformed into chemical energy contained in organic material by the photosynthesis process. In photosynthesis light, water and carbon dioxide react and highly energetic glucose molecules and molecular oxygen are made.		Behavior-2
What are the stages into which photosynthesis is divided?	Light reaction and Calvin cycle	What are the 2 stages of photosynthesis?	Structure
What are the processes of the photochemical stage of the photosynthesis process?	cyclic photophosphorylation and noncyclic photophosphorylation	What are the 2 stages of the light reaction in photosynthesis?	Structure
What is NADP and NADPH?	NADP is the abbreviation		Structure:

-structure	of the nicotinamide adenine dinucleotide phosphate cation. NADPH is made when NADP binds to one hydrogen atom.		boundary
What is NADP and NADPH?<br>-function	NADP is a hydrogen acceptor. NADPH is the form that actually transports hydrogen.		Function
Which chemical element is central in the chlorophyll molecule?	The chemical element that is central in the chlorophyll molecule is magnesium. One atom of magnesium is present in the center of an amalgam of eight nitrogen-containing carbon rings.		Structure
In which chloroplast structure are chlorophyll molecules found?	Chlorophyll molecules sit on the surface of each thylakoid		Structure: boundary
In photosynthesis, what is the molecule that donates hydrogen for photosynthesis?	H2O		Function
Photosynthesis is the most important producer of molecular oxygen (O2) on our planet. From which molecule do oxygen atoms liberated by	The oxygen atoms liberated as molecular oxygen by the photosynthesis process come from water.		Behavior-2

photosynthesis come? From which other molecule could one suspect they have come? What are the destinations of those oxygen atoms?	One indeed could suspect that those oxygen atoms would have come from carbon dioxide. Oxygen atoms from carbon dioxide however are incorporated into glucose molecules and into water molecules liberated in the chemical stage of photosynthesis.		
Where do the photochemical and the chemical stages of photosynthesis occur?	Stroma of chloroplast	Where does the calvin cycle of photosynthesis occur?	Structure
What is the destination of each of those substances produced by water photosynthesis?	The electrons will replace those electrons lost by chlorophyll molecules in photophosphorylation. The hydrogen ions will be incorporated into hydrogen acceptor molecules (NADP) and later will be used in the synthesis of glucose during the chemical stage. Molecular oxygen is liberated to the atmosphere.		Behavior-2
How is the photic energy	Light excites chlorophyll		Behavior-2

absorbed by chlorophyll transfered to ATP molecules in photophosphorylation?	and energizes electrons that jump off the molecule. The energy liberated when these electrons escape is used in the phosphorylation of ADP, forming ATP. The enzyme that catalyzes the reaction is the ATP synthase.		
How will be the resulting ATP of photophosphorylation used?	The resulting ATP is then consumed in the next chemical stage of photosynthesis to energetically enrich carbon dioxide for the formation of glucose.		Behavior-2
What are the chemical substances produced by water photolysis?	Free electrons, hydrogen ions and molecular oxygen are liberated, after the water photolysis.		Behavior-1
How are the large number of ATP and NADPH molecules used during the Calvin cycle consistent with the high value of glucose as an energy source?	Glucose can be used by plants as food or structure and so without it a plant would die. The investment of so much ATP and NADPH is worth living		Behavior-2

	for.		
In the light actions, what is the initial electron donor? Where do the electrons finally end up?	Water (H2O) is the initial electron donor; NADP+ accepts electrons at the end of the electron transport chain, becoming reduced to NADPH.	Please explain the process of noncyclic photophosphorylation in the light reaction.	Structure
Which are the living beings that carry out photosynthesis?	Plants, algae and cyanobacteria are photosynthetic beings.		Function
Which is the cell organelle responsible for the absorption of light for the photosynthesis process in plants and algae?	Light is absorbed by chlorophyll, a molecule present in cytoplasmic organelles called chloroplasts.		Function
What are the roles of ATP and ADP for the cellular energetic metabolism?	The conversion between ATP, and ADP and phosphate, plays a central role in the energy metabolism of the cell.		Function
Why is it said that during photosynthesis carbon dioxide is enriched to form glucose?	During photosynthesis carbon dioxide is energetically enriched with hydrogen from water. Water broken by photolysis is the hydrogen donor of the reaction. Glucose is made of carbon		Function

	and oxygen atoms obtained from carbon dioxide and of hydrogen atoms obtained from water.		
What are the roles of NADPH and ATP in the chemical stage of photosynthesis?	NADPH acts as reductant of carbon dioxide, it delivers highly energetic hydrogens to precursor molecules during the glucose formation process. ATP is an energy source for the reactions of chemical stage.	In the calvin cycle of photosynthesis, what is the role of atp?<br>What does the nadph do in the reduction of 3 phosphoglycerate of calvin cycle?	Function
Why is the carbon dioxide concentration a limiting factor of the photosynthesis process?	The availability of carbon dioxide is a limiting factor for the photosynthesis process because this gas is a reagent of the reaction.	How is the carbon dioxide used in the carbon fixation of calvin cycle?	Behavior-2
When the carbon dioxide concentration is increased indefinitely, is photosynthesis also increased indefinitely?	Since enzymes catalyze the building of organic molecules with carbon atoms from carbon dioxide photosynthesis stops as soon as these enzymes become saturated, i.e., when all their activation centers are bound to their substrates. In that situation		Behavior-2

	an increase of the carbon dioxide concentration will not increase the photosynthesis rate.		
Explain why a poison that inhibits an enzyme of the Calvin cycle will also inhibit the light reaction	The light reactions require ADP and NADP+, which would not be formed in sufficient quantities from ATP and NADPH if the Calvin cycle stopped.	How does the light reaction support the calvin cycle?	Function
